# Supplementary material for: Reduced MIP-1β as a Trait Marker and Reduced IL-7 and IL-12 as State Markers of Anorexia Nervosa
Source: J Pers Med. 2021 Aug 20;11(8):814. doi: 10.3390/jpm11080814 (PMC8399452; doi:10.3390/jpm11080814)
Supplement: Supplementary file 1 [file jpm-11-00814-s001.zip › jpm-1328568-supplementary.pdf]

Table S1. Metrics of inflammatory markers in the whole sample.

Inflammatory marker	Undetectable Below fit curve (n (%))	Low detection limit (pg/ml)	High detection limit (pg/ml)
Eotaxin	0 (0%)	2.29	1880
Eotaxin-3	0 (0%)	0.692	6240
GM-CSF	31 (22%)	0.116	1260
IFN-γ	0 (0%)	0.215	1500
IL-1α	79 (56%)	0.920	454
IL-1β	110 (77%)	0.064	610
IL-2	8 (6%)	0.042	1440
IL-4	46 (32%)	0.015	218
IL-5	112 (79%)	0.190	857
IL-6	0 (0%)	0.061	742
IL-7	0 (0%)	0.116	830
IL-8	0 (0%)	0.032	612
IL-10	0 (0%)	0.020	378
IL-12/IL-23p40	0 (0%)	0.250	3650
IL-12p70	17 (12%)	0.066	549
IL-13	25 (18%)	0.721	529
IL-15	0 (0%)	0.142	941
IL-16	0 (0%)	0.212	2910
IL-17α	0 (0%)	0.256	6240
IL-21	102 (72%)	0.681	1180
IL-22	6 (4%)	0.216	542
IL-23	131 (92%)	0.455	5540
IL-27	0 (0%)	12.9	18200
IL-31	46 (32%)	0.070	964
IP-10	0 (0%)	0.169	2360
MCP-1	0 (0%)	0.035	562
MCP-4	0 (0%)	4.24	724
MDC	0 (0%)	3.34	11800
MIP- 3α	2 (1%)	0.277	566
MIP-1α	8 (6%)	3.54	11800
MIP-1β	0 (0%)	0.803	1270
TARC	0 (0%)	0.310	1800
TNF-α	0 (0%)	0.118	367

TNF-β	17 (12%)	0.080	711
VEGF	0 (0%)	0.326	1350

Abbreviations: IFN = interferon; IL = interleukin; IP = interferon γ-induced protein; MCP = monocyte chemoattractant protein; MDC = macrophage-derived chemokine; MIP = macrophage inflammatory protein; TARC = thymus and activation-regulated chemokine; TNF = tumor necrosis factor; VEGF = vascular endothelial growth factor.
